# Hygiene

**Published:** 1882-01

**Authors:** 


### HYGIENE,

PRONOUNCED hygeen, with two syllables, is from the name of Hygieia, who was the daughter of Æsculapius. There were statues erected to her as the goddess of health. Her father, according to the mythology of the Greeks, was the god of health; he was the son of Apollo, who was the model of manly beauty, the god of medicine, music, poetry and eloquence. He brought up his son to the study of medicine and to hunting. There was always a meaning in these old Greek legends, and the mind naturally connects hunting and its necessary activities in the open air, its exhilarations and its excitements with health—health without medicine, as if Apollo thought that if health could be maintained and regained without the use of medicine, it was the perfection of the healing art; and now, three thousand years later, the people think the same thing, and this is the idea connected with the word hygiene, the maintenance of health and the recovery from disease by other means than medicine, such as judicious attention to air and exercise, to dress, clothing, eating, sleeping and the general habits of life.—*Dr. Hall's Health at Home.*

